# Digital Health and Self-Management in Idiopathic Inflammatory Myopathies: A Missed Opportunity?

**DOI:** 10.1007/s11926-024-01157-6

**Published:** 2024-08-08

**Authors:** Simone Battista, Benedetto Giardulli, Cristiana Sieiro Santos, Or Aharonov, Darshan Puttaswamy, Anne – Marie Russell, Latika Gupta

**Affiliations:** 1 School of Health and Society, Centre for Human Movement and Rehabilitation, University of Salford, Salford, Greater Manchester, UK; 2 Department of Neurosciences, Rehabilitation, Ophthalmology, Genetics, Maternal and Child Health, University of Genova, Campus of Savona, Savona, Italy; 3 Department of Rheumatology, Complejo Asistencial Universitario de León, León, Spain; 4 Department of Psychology, Psychology of Aging Group, University of Vienna, Vienna, Austria; 5 Seth Gordhandhas Sunderdas Medical College and King Edward Memorial Hospital, Mumbai, Maharashtra India; 6 School of Medicine and Health, University of Birmingham, Edgbaston, Birmingham, UK; 7 School of Health and Care Professions, University of Exeter, Exeter, Devon, UK; 8 ILD Regional Service, University Hospitals Birmingham NHS Trust, Birmingham, UK; 9 Department of Rheumatology, Royal Wolverhampton Hospitals NHS Trust, Wolverhampton, UK; 10 Division of Musculoskeletal and Dermatological Sciences, Centre for Musculoskeletal Research, School of Biological Sciences, Faculty of Biology, Medicine and Health, Manchester Academic Health Science Centre, The University of Manchester, Manchester, UK

**Keywords:** Rheumatic diseases, Digital health, Patient education as topic, Health knowledge, Attitudes, Practice, Self-management

## Abstract

**Purpose of Review:**

This paper explored the potential of digital health in idiopathic inflammatory myopathies (IIMs), with a focus on self-management. Digital self-management technology includes tailored treatment plans, symptom tracking, educational resources, enhanced communication, and support for long-term planning.

**Recent Findings:**

After arguing the importance of digital health in IIMs management, from diagnosis until treatment, our literature review revealed a notable gap in research focusing on the efficacy of digital self-management interventions for individuals with IIMs, with no randomised controlled trials or observational studies addressing this topic.

**Summary:**

Our review further highlighted the significant unmet need for research in self-management interventions for individuals with IIMs. The absence of studies underscores the necessity for collaborative efforts to address this gap and develop personalised, effective strategies for managing IIMs using digital technology. Individuals with IIMs deserve tailored self-management approaches akin to those available for other rheumatic and musculoskeletal diseases.

**Supplementary Information:**

The online version contains supplementary material available at 10.1007/s11926-024-01157-6.

## Introduction

Idiopathic inflammatory myopathies (IIMs), also known as myositis, are heterogeneous disorders associated with chronic inflammation of the skeletal muscles, varying in their clinical manifestations, treatment responses, and prognoses [1]. Muscle weakness is usually the predominant clinical manifestation. Nevertheless, IIM often impacts other organs, such as the skin, joints, lungs, heart, and gastrointestinal tract, which may emerge as predominant manifestations [1]. Consequently, healthcare professionals from inter/multidisciplinary specialities are engaged in the comprehensive care and management of individuals with IIMs [2].

### The Challenges of IIMs for Health Professionals and Patients

IIMs present a complex array of challenges for both healthcare professionals and patients. Firstly, skeletal muscle inflammation in IIMs often leads to impaired mobility, pain, and fatigue. These symptoms can affect an individual’s motivation to engage in healthcare and cause irregularities in symptom monitoring and management [2]. Secondly, the rarity of IIMs contributes to limited evidence on management approaches, resulting in a lack of awareness and knowledge among healthcare professionals. These considerations can lead to misdiagnosis, delays in treatment, access to specialist care and increased disease severity [3, 4]. Thirdly, individuals with IIMs often have restricted access to information and support resources. This lack of access can exacerbate feelings of isolation and uncertainty, which may, in turn, increase anxiety [4]. Addressing these challenges requires innovative approaches to manage better the complexities associated with IIMs.

### Digital Healthcare and Self-Management in IIMs: A Promising Solution

Recently, remarkable technological advancements have opened several opportunities to help all the stakeholders in rheumatology healthcare [5]. Utilising digital health to manage rheumatological conditions offers unique opportunities [6]. Digital healthcare involves applying information and communication technologies in healthcare to manage health risks and illnesses while promoting overall wellness [7]. Its goal is to enhance care quality by addressing inefficiencies, providing more equitable and accessible healthcare, reducing the burden of travel, increasing healthcare accessibility, and delivering tailored healthcare to individuals [7]. Digital health encompasses various technologies, including health information technology, wearable devices, mobile health apps, and telemedicine [5, 8]. It covers electronic health records, virtual visits, digital therapeutics and self-management [5, 8], with its usage particularly flourishing during and after the COVID-19 pandemic [9].

Barlow defined self-management as “the individual’s ability to manage the symptoms, treatment, physical and psychosocial consequences and the lifestyle changes inherent in living with a chronic condition” [10]. This approach involves collaborative efforts between healthcare professionals and patients that can be done face-to-face or online with digital health technologies [2]. Self-management strategies have been of interest for many years among individuals living with long-term conditions. A notable paradigm shift emphasises the patient’s key role in care management to reach a shared decision-making approach, with greater responsibility for their care in partnership with healthcare professionals [11]. Self-management encompasses patient education, awareness of their illness and health beliefs, knowledge to track and monitor symptoms, attitude and behaviour towards lifestyle modification, and a focus on promoting social and psychological well-being [12]. The Institute of Medicine (IOM), sponsored by the Arthritis Foundation and the Centre for Disease Control and Prevention (CDC), has produced a report titled “Living Well with Chronic Illness: A Call for Public Health Action” [13]. This document reported self-management as a crucial framework for health and well-being, fostering behavioural change in patients towards their illness and providing them with the knowledge and skills to care for themselves and improve their health outcomes [13]. Corbin and Strauss identified three sets of tasks associated with self-managing chronic illness: (1) medical management of the condition, (2) behaviour management, and (3) emotional management. Later, five core practices were defined, including problem-solving, decision-making, resource utilisation, partnerships with healthcare professionals, and taking action [14]. E-health solutions like telemedicine or teleconsultation can facilitate self-management [14].

In IIMs, self-management is paramount, considering the absence of disease-specific interventions [2]. The core of self-management in IIMs lies in changing how people with IIMs deal with their symptoms, incorporating exercise, comorbidity management, and psychosocial interventions that foster emotional support in their daily self-care [2, 13]. The management of individuals with IIMs encompasses three crucial areas. Firstly, conservative care is essential to minimise the impact of weakness on joints, bones, and other systems. Secondly, it is fundamental to address comorbidities associated with the disease. Thirdly, there is a need to maximise individuals’ functional abilities and quality of life (QoL) [15]. King & Kissel have introduced a multimodal approach with four intervention categories for the self-management of people with muscle diseases [15]. These categories include (1) Strength therapies; (2) Supportive care targeting issues resulting from muscle weakness; (3) Symptomatic care addressing problems inherent to individuals’ conditions but not directly related to muscle disease; and (4) Psychological support to enhance individuals’ mental outlook and provide information about their diseases [16].

The application of digital health in IIMs self-management can be advantageous in the following areas of the model:

1. *Tailored Treatment Plans*: Self-management technology can assist health professionals in creating personalised treatment plans. The technology can help to collect data on individuals’ symptoms to tailor interventions based on individual needs, aligning with the first category of the IIM multimodal approach aimed at improving strength [10, 15, 16];
2. *Remote Monitoring and Adherence*: Health professionals can effectively conduct remote consultations. While surgeons may encounter certain limitations with teleconsultations, these platforms remain valuable for pre-operative and post-operative care. [17]. Self-management digital technologies offer remote monitoring capabilities, tracking individuals’ progress and therapy adherence. This is particularly relevant to addressing problems resulting from muscle weakness, as it enables prompt intervention when signs of deterioration appear. [18, 19]. A recent study by Ahmed et al. found that more than 70% of rheumatology consultations could be handled remotely [19]. Wearable devices like Actigraph and fitness trackers offer passive monitoring, providing data indicating significant changes in muscle strength and making people aware of their activity levels [20].
3. *Symptom Management*: Self-management technologies are valuable assets for individuals dealing with IIMs, extending beyond the direct concerns related to muscle disease (the third category of the IIM multimodal approach). For instance, pain-tracking apps empower users to monitor and communicate their pain levels effectively. This facilitates more accurate discussions with healthcare professionals and leads to improved symptom management [19]. In addition, patient-reported outcomes and novel self-performed tests, like the 10-times arm-lift test, offer alternative approaches to traditional methods, such as Manual Muscle Testing (MMT8), enabling remote monitoring of muscle strength in individuals with myositis. A notable example supporting the efficacy of mobile health (mHealth) apps comes from a 2022 multicentric study by Fedkov et al., which showcased improvements in health-related quality of life and disease activity in various inflammatory conditions [21–23]. Although myositis-specific mobile applications like MioApp primarily cater to physicians, the potential for more patient-centric apps is vast. Customised apps could provide disease information in a user-friendly format, offer reliable insights, facilitate symptom monitoring, and include features for medical assistance and connectivity with healthcare professionals. This unexplored avenue holds significant promise for enhancing the experience of people with IIMs and warrants further exploration [24–26].
4. *Education and Empowerment*: Digital self-management technology is pivotal in delivering educational resources to individuals managing IIMs, fostering a comprehensive understanding of their condition. A cross-sectional study in Saudi Arabia revealed that over 50% of healthcare professionals recognise the benefits of integrating social networking sites into healthcare services, promoting individual education and public health awareness [19, 27]. This empowerment enables individuals to actively engage in their care, contributing to informed decision-making and mitigating the power imbalance between healthcare professionals and patients [28–30]. Social media, especially for rare diseases like IIMs, emerges as a potent tool for building communities, fostering mutual support, and sharing valuable experiences [31]. Studies evaluating myositis-related content on YouTube emphasised the importance of specialist involvement in developing medically related videos using validated tools to ensure accurate health information dissemination [19, 32–35]. However, a recent study identified misinformation on rare diseases from specific online resources, reiterating the need for credible sources of information and establishing pathways to signpost individuals to these [34].

Social media, while valuable, can be a double-edged sword, underscoring the imperative of averting the ‘spiral of silence,’ a phenomenon wherein individuals withhold their opinions or concerns when they believe they are in the minority, apprehensive of facing isolation, backlash or feeling marginalised within online communities [36]. Social media dynamics may vary by region, highlighting the significance of a global partnership and establishing regional patient support groups [37]. Striking a balance between the benefits and drawbacks of digital platforms is crucial to ensure a positive and empowering experience for individuals managing myositis in the digital age. Additionally, the landscape suggests a need for a greater partnership between Patient Research Partners (PRPs) and leaders who train the next generation of PRPs [38–43]. Patients often contribute to generating valid and reliable information sources easily understandable by the lay public to advance self-management [38–43]. The patient’s role in research, exemplified by the COVID-19 Vaccination in Autoimmune Diseases (COVAD) study, has significantly advanced the patient voice in the realm of rare disease research [44–47]. This study has played a pivotal role in bringing meaningful contributions to the literature, aiming to bridge the gap between patient and clinician disparities in reporting. Patient-reported outcomes (PROs) are gaining increasing importance in the specific context of myositis, particularly as self-management practices become more prevalent. Validating PROs becomes crucial for accurate representation of self-report both at individual and population levels [48]. The global consortium of the COVAD study has delved into the dispersion of variables and employed triangulation with various levels of physical function, providing valuable insights. It sheds light on the patient experience of fatigue, emphasising the importance of advocacy, self-empowerment, and active involvement in research to inform future research directions and clinical care [49]. Considering QoL, it becomes evident that various variables have a significant impact. Self-management practices necessitate a deeper understanding of these variables for effective patient care. In this evolving landscape, patient involvement and empowerment play a pivotal role in shaping the narrative of their experience and contributing valuable perspectives that enrich the research and clinical care landscape.

5. *Communication and Collaboration*: Self-management technology stands as a catalyst for enhanced communication between healthcare professionals and patients with IIMs, fostering collaborative and effective healthcare partnerships. Research demonstrates that electronic communication supplements traditional methods, reinforcing health professionals’ advice and improving adherence among those with long-term conditions [22]. Despite concerns about limited physical examinations and the need for additional tests, patients exhibit acceptance of teleconsultations [50–53]. The pandemic and the sequelae of long-COVID have triggered a rapid digital transformation in medicine, manifesting in the widespread adoption of teleconsultations and the utilisation of digital tools for remote monitoring. Telemedicine has proven beneficial for individuals dealing with rheumatologic diseases (like IIMs), eliminating the need for frequent hospital visits and reducing the burden on healthcare systems, especially in resource-constrained regions [25]. Notwithstanding these advantages, Naveen et al. delved into various challenges inherent in teleconsultations, encompassing mental health concerns, overlooked symptoms, language barriers, privacy issues, and technological hurdles [53]. The authors also put forth solutions and remedial measures to mitigate these obstacles [53]. Further, integrating digital tools and patient-initiated care models is reshaping the communication landscape of healthcare. This transformation has been facilitated by the growing popularity of social media among health professionals, with approximately 60% preferring its use for interaction with patients [26]. This preference is driven by the belief that leveraging social media can improve education, compliance, and outcomes [26]. The patient’s perspective also reflects the paradigm shift toward integrating social media into healthcare. A survey conducted in an outpatient family practice clinic revealed that 56% of patients desired their healthcare professionals to utilise social media for various purposes, including communication for appointment reminders, test results, prescriptions, and addressing general questions [54]. Even individuals not currently using social media expressed willingness to start if it facilitated connection with their healthcare professionals [54]. This evolving landscape underscores the potential of self-management technology and digital platforms to optimise myositis communication, engagement, and healthcare outcomes.

6. *Long-term Planning*: Self-management technology can assist health professionals in tracking the progression of the disease over time, aiding in adjusting treatment strategies as needed, as IIMs as chronic conditions require long-term planning. Integrating self-management technology is instrumental in facilitating long-term planning for disease management [55, 56]. These technologies enable continuous monitoring of critical indicators such as muscle strength, activity levels, and symptoms, providing a dynamic and real-time understanding of the disease’s progression. Tracking these parameters over time empowers healthcare professionals to make informed decisions regarding treatment adjustments, ensuring a personalised and responsive approach to the evolving nature of IIMs. Moreover, self-management technology supports long-term planning by promoting medication adherence through features like medication tracking and reminders [57, 58]. It facilitates remote consultations, allowing individuals to engage in regular virtual check-ins with healthcare professionals, fostering ongoing assessments and adjustments to treatment strategies. The wealth of data generated by these tools contributes to data-driven decision-making, identifying correlations between lifestyle factors and disease activity. Ultimately, the patient-centric nature of self-management technology empowers individuals to participate actively in their care, promoting a collaborative and proactive approach to long-term well-being in managing myositis [22].

## Limitations of Digital Healthcare

The rise of digital technologies has undoubtedly revolutionised access to information, offering unprecedented opportunities for individuals to educate themselves about various topics, including healthcare. However, despite this advancement, a significant portion of the population still grapples with limited digital literacy skills, creating barriers to accessing and understanding crucial health information [59]. This challenge is particularly pronounced among underserved communities, who already face disparities in healthcare access due to factors such as mistrust in healthcare systems, rural living, socioeconomic deprivation, and language barriers [60]. For these individuals, navigating digital platforms for health information can be daunting and overwhelming [61].

Further, the influence of social media in disseminating health information cannot be overstated. While social media platforms can serve as valuable tools for connecting individuals, sharing experiences, and accessing support networks, they also present risks. One such risk discussed above is the ‘spiral of silence’ phenomenon. This phenomenon can stifle open dialogue and impede the exchange of valuable health information, particularly on sensitive or controversial topics [40]. Recognising that social media dynamics may vary significantly by region, culture, and community norms is essential. Therefore, fostering a global partnership and establishing regional patient support groups can mitigate the harmful effects of the spiral of silence and promote open communication and collaboration in healthcare discussions.

In summary, while digital technologies and social media platforms offer immense potential for improving healthcare access and communication, addressing the challenges they pose is vital, including limited digital literacy and the risk of the spiral of silence [40, 59]. Acknowledging these issues and advocating for tailored interventions and global partnerships, we can work towards ensuring equitable access to accurate health information and resources for everyone, regardless of their digital literacy skills or social media presence.

### Digital Self-Management in IIMs: What Do We Know from the Literature?

EULAR 2021 highlighted using mobile health applications (apps) and digital healthcare in patients with rheumatic and musculoskeletal diseases to better self-manage their health and improve clinical outcomes [58]. Many individuals with rheumatic and musculoskeletal diseases have been shown to use a smartphone regularly (91%). Studies reveal that people with these conditions use mobile health technologies to improve their disease status and track symptoms and disease activity [50, 51]. So, using e-health for self-management in these individuals is a promising mode for symptom management and better outcomes.

Considering the potential roles of digital self-management in IIMs, we run a narrative review to gain insights from the literature about studies investigating the effectiveness of self-management strategies in IIMs (either digitally or not) in improving individuals’ symptoms, functionality, and quality of life. We conducted this review on all kinds of self-management strategies in IIMs as some studies might not be explicit that the self-management was digital in their title/abstract. We followed Gasparyan et al.’s narrative biomedical review guidelines [62]. We searched Medline via PubMed and EMBASE until 23 June 2023 for randomized controlled trials or longitudinal observational studies with at least two arms (case and control) evaluating self-management strategies in IIMs. We used the abovementioned definition of Barlow et al. to refer to self-management [10]. Our primary outcomes were pain, disability and functionality (e.g., activity of daily life). Secondary outcomes were quality of life and psychological outcomes.

**Fig. 1:**
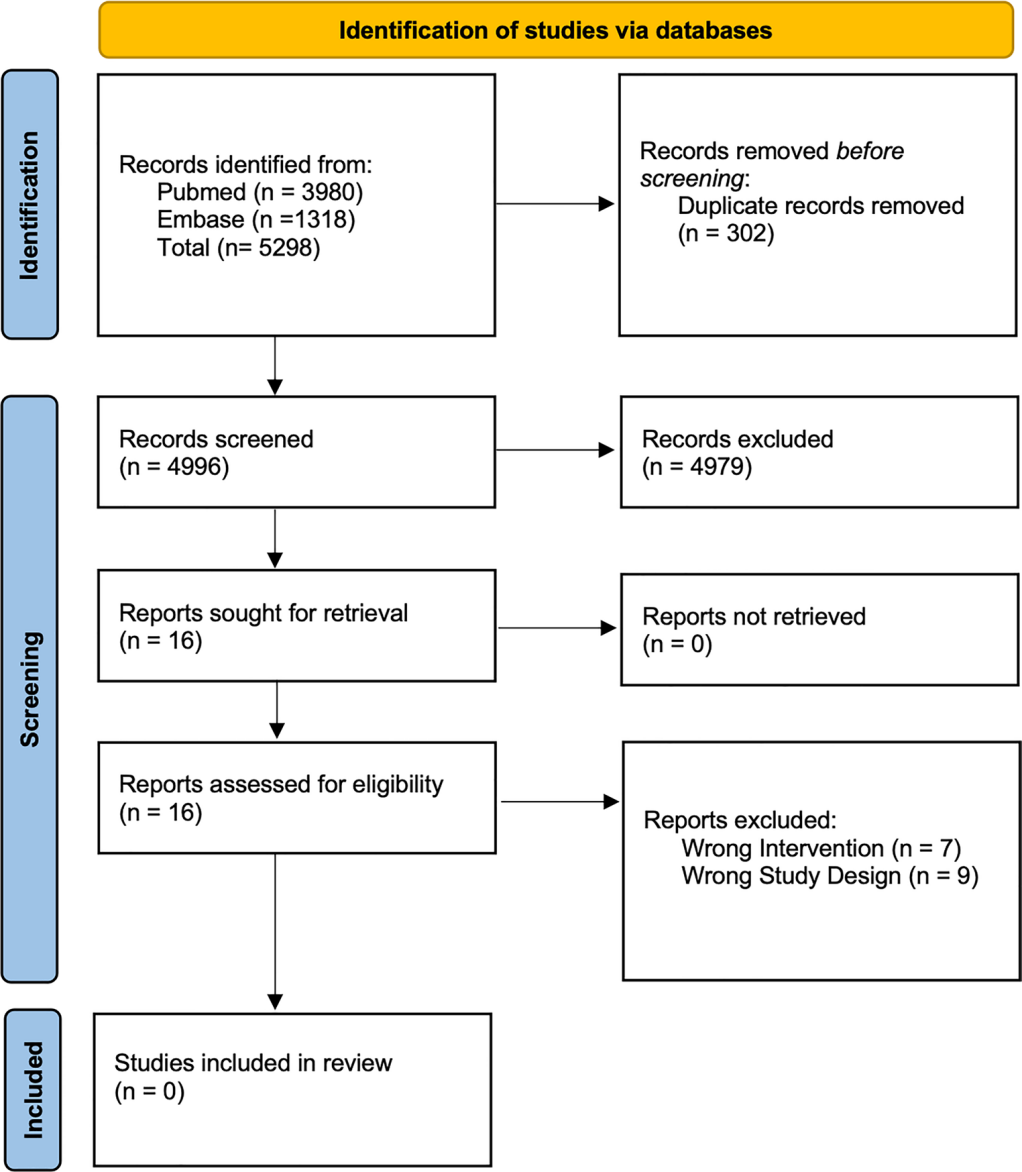
Prisma Flow Diagram of the included studies

To be included, studies had to compare the effectiveness of self-management strategies against other treatments, no treatments, or in addition to other therapies. No limits on the age population were set. We only looked for papers written in English after 2000. We did not specify any inclusion/exclusion criteria for the outcomes. The search was conducted by two authors (SB and BG). The complete search strategy is in the appendix (Supplementary Material 1). Articles obtained from the research were uploaded to the Covidence Systematic Review Software (Covidence systematic review software, Veritas Health Innovation, Melbourne, Australia. Available at www.covidence.org*).* After duplicate removal, two independent authors (BG and DP) selected the studies applying the inclusion and exclusion criteria by reading the titles and abstracts. Then, they also read the full texts of the included studies. A third author (SB) was consulted in case of disagreement. We started with *n* = 5,298 studies. Covidence removed *n* = 302 studies as they were duplicates. Therefore, *n* = 4,996 studies were evaluated for title and abstract screening. We had 16 full texts to read, and none answered our research questions. Consequently, we did not find any RCTs or observational longitudinal studies testing the effectiveness of self-management strategies (either online or face-to-face) in IIMs. Supplementary Material 2 provides the 16 studies and reasons for exclusion. See the PRISMA flow diagram of the study selection (Fig. 1). In line with our results, though online self-management strategies can be helpful in IIMs, there is still an unmet need for these interventions in IIMs [2].

## Discussion

In rheumatology, recent technological advancements have paved the way for unique opportunities in healthcare [5, 29]. Conditions such as osteoarthritis and inflammatory arthritis have seen substantial research efforts dedicated to patient education, symptom monitoring, and overall disease management [5, 6, 30]. Such advancements have significantly advanced education and digital interventions for various rheumatic and musculoskeletal diseases [58]. However, IIMs remain relatively underserved in terms of self-management strategies. The literature review highlighted a critical gap in studies evaluating digital or non-digital self-management strategies for individuals with IIMs. While digital healthcare in IIMs has focused on diagnosis, consultation and monitoring, there is a pressing need for digitally supported self-management interventions. Telehealth solutions in IIMs might offer accessible and patient-centric approaches, including voice consultations and remote monitoring [30]. Wearables, video consultations, and electronic patient-reported outcomes facilitate continuous and passive monitoring of disease activity, providing a complementary perspective to traditional assessment [30]. These technologies address the challenges of fluctuating IIM disease activity and provide a more holistic understanding of patients’ conditions [30]. Individuals with IIMs deserve the same level of attention and innovation also in self-management strategies as their counterparts with other rheumatic and musculoskeletal disorders (RMD). The unmet need for digital self-management interventions in IIMs is a pressing concern that warrants immediate attention from researchers, healthcare professionals, and policymakers. Their concerted efforts will ultimately integrate digital healthcare solutions into the care of IIM patients, making these interventions more widely accepted and open for exploration. [2]. Although existing evidence is limited, it indicates that patients can be adequately and safely supported through digital means throughout their healthcare journey [12, 63]. The growing interest in digital healthcare technologies promises to improve the overall management and outcomes for individuals with IIMs. Hence, randomised controlled trials testing the efficacy of self-management interventions’ (digital or not) are necessary.

## Conclusion

Recognising and addressing the unmet need for self-management approaches in IIMs is crucial. Integrating digital healthcare and self-management technologies offers a promising approach to tackling the challenges of managing IIMs. These technologies provide tailored solutions that enhance patient engagement, education, and well-being. Embracing the potential of digital self-management interventions presents an opportunity to usher in a new era of improved care and enhanced quality of life for individuals with IIMs. Further research and concerted efforts are essential to ensure that individuals with IIMs receive comprehensive, tailored care aligned with the evolving landscape of digital healthcare.

## Electronic Supplementary Material

Below is the link to the electronic supplementary material.

Supplementary Material 1

Supplementary Material 2

## Data Availability

No datasets were generated or analysed during the current study.

## References

[1] Idiopathic inflammatory myopathies. Nat Rev Dis Primers. 2021;7:87. 34857780 10.1038/s41572-021-00325-7 PMC10425161

[2] Gupta L, Deshmukh P, Thornton C, Aggarwal R, Nikiphorou E. Addressing the unmet need for self-management strategies in idiopathic inflammatory myositis. RMD Open. 2023;9. 10.1136/rmdopen-2022-002745 PMC9923345 36764703

[3] Bhai SF, Dimachkie MM, de Visser M. Is it really myositis? Mimics and pitfalls. Best Pract Res Clin Rheumatol. 2022;36. 10.1016/j.berh.2022.101764 35752578

[4] Lilleker JB, Gordon P, Lamb JA, Lempp H, Cooper RG, Roberts ME et al. Patient-centred standards of care for adults with myositis. BMC Rheumatol. 2017;1. 10.1186/s41927-017-0002-7 PMC6383593 30886948

[5] Solomon DH, Rudin RS. Digital health technologies: opportunities and challenges in rheumatology. Nat Rev Rheumatol. 2020;16:525–35. 32709998 10.1038/s41584-020-0461-x

[6] de Thurah A, Bosch P, Marques A, Meissner Y, Mukhtyar CB, Knitza J, et al. 2022 EULAR points to consider for remote care in rheumatic and musculoskeletal diseases. Ann Rheum Dis. 2022;81:1065–71. 35470160 10.1136/annrheumdis-2022-222341

[7] Ronquillo Y, Meyers A, Korvek SJ. Digital healthcare. In: StatPearls [Internet]. Treasure Island (FL): StatPearls Publishing; 2023.

[8] Kuwabara A, Su S, Krauss J. Utilizing Digital Health Technologies for Patient Education in Lifestyle Medicine. Am J Lifestyle Med. 2020;14:137. 32231478 10.1177/1559827619892547 PMC7092400

[9] Gupta L, Najm A, Kabir K, De Cock D. Digital health in musculoskeletal care: where are we heading? BMC Musculoskelet Disord. 2023;24. 10.1186/s12891-023-06309-w PMC10012296 36918856

[10] Barlow J, Wright C, Sheasby J, Turner A, Hainsworth J. Self-management approaches for people with chronic conditions: a review. Patient Educ Couns. 2002;48:177–87. 12401421 10.1016/s0738-3991(02)00032-0

[11] Morrison T, Foster E, Dougherty J, Barton J. Shared decision making in rheumatology: a scoping review. Semin Arthritis Rheum. 2022;56. 10.1016/j.semarthrit.2022.152041 35738040

[12] Knitza J, Kuhn S, Gupta L. Digital Approaches for Myositis. Curr Rheumatol Rep. 2023;25:259–63. 37962833 10.1007/s11926-023-01119-4 PMC10754733

[13] Grady PA, Gough LL. Self-management: a comprehensive approach to management of chronic conditions. Am J Public Health. 2014;104:e25–31. 24922170 10.2105/AJPH.2014.302041 PMC4103232

[14] Harris JR, Wallace RB. The Institute of Medicine’s new report on living well with chronic illness. Prev Chronic Dis. 2012;9:E148. 22995102 10.5888/pcd9.120126 PMC3475533

[15] Schulman-Green D, Jaser S, Martin F, Alonzo A, Grey M, McCorkle R, et al. Processes of self-management in chronic illness. J Nurs Scholarsh. 2012;44:136–44. 22551013 10.1111/j.1547-5069.2012.01444.x PMC3366425

[16] Gupta L, Najm A, Kabir K, De Cock D. Digital health in musculoskeletal care: where are we heading? BMC Musculoskelet Disord. 2023;24:192. 36918856 10.1186/s12891-023-06309-w PMC10012296

[17] Joshi M, Jagtap RN, Gupta K, Agarwal R, Aggarwal V. Assessment of quality and reliability of YouTube videos for patient and physician education on inflammatory myositis. Clin Rheumatol. 2023;42:1339–49. 36759401 10.1007/s10067-023-06522-x PMC9910767

[18] King M, Kissel W. Multidisciplinary approach to the management of myopathies. Continuum (Minneap Minn). 2013;19:1650–73. 6 Muscle Disease. 24305452 10.1212/01.CON.0000440664.34051.4d PMC4234135

[19] Ahmed S, Kelly YP, Behera TR, Zelen MH, Kuye I, Blakey R, et al. Utility, appropriateness, and content of Electronic consultations Across Medical subspecialties. Ann Intern Med. 2020;172:641–7. 32283548 10.7326/M19-3852

[20] Chretien KC, Kind T. Social media and clinical care: ethical, professional, and social implications. Circulation. 2013;127:1413–21. 23547180 10.1161/CIRCULATIONAHA.112.128017

[21] Gamwell KL, Kollin SR, Gibler RC, Bedree H, Bieniak KH, Jagpal A, et al. Systematic evaluation of commercially available pain management apps examining behavior change techniques. Pain. 2021;162:856–65. 33003110 10.1097/j.pain.0000000000002090 PMC9152920

[22] Fedkov D, Berghofen A, Weiss C, Peine C, Lang F, Knitza J, et al. Efficacy and safety of a mobile app intervention in patients with inflammatory arthritis: a prospective pilot study. Rheumatol Int. 2022;42:2177–90. 36112186 10.1007/s00296-022-05175-4 PMC9483251

[23] Kwan YH, Ong WJ, Xiong M, Leung YY, Phang JK, Wang CTM, et al. Evaluation of mobile apps targeted at patients with Spondyloarthritis for Disease Monitoring: systematic app search. JMIR Mhealth Uhealth. 2019;7:e14753. 31661080 10.2196/14753 PMC6913729

[24] Shaw Y, Courvoisier DS, Scherer A, Ciurea A, Lehmann T, Jaeger VK et al. Impact of assessing patient-reported outcomes with mobile apps on patient-provider interaction. RMD Open. 2021;7. 10.1136/rmdopen-2021-001566 PMC8023945 33811177

[25] Romero-Jimenez R, Escudero-Vilaplana V, Chamorro-De-Vega E, Ais-Larisgoitia A, Lobato Matilla ME, Herranz-Alonso A, et al. The characteristics and functionalities of mobile apps aimed at patients diagnosed with Immune-mediated inflammatory diseases: systematic app search. J Med Internet Res. 2022;24:e31016. 35254286 10.2196/31016 PMC8933793

[26] Alshakhs F, Alanzi T. The evolving role of social media in health-care delivery: measuring the perception of health-care professionals in Eastern Saudi Arabia. J Multidiscip Healthc. 2018;11:473–9. 30275699 10.2147/JMDH.S171538 PMC6157575

[27] Wakefield D, Bayly J, Selman LE, Firth AM, Higginson IJ, Murtagh FE. Patient empowerment, what does it mean for adults in the advanced stages of a life-limiting illness: a systematic review using critical interpretive synthesis. Palliat Med. 2018;32:1288–304. 29956568 10.1177/0269216318783919 PMC6088522

[28] Yang H, Guo X, Peng Z, Lai KH. Patient empowerment in an online health platform: exploring the quadratic effects of patients’ conscious-competence on perceived health status. Comput Hum Behav. 2022;136:107346.

[29] Madanian S, Nakarada-Kordic I, Reay S, Chetty T. Patients’ perspectives on digital health tools. PEC Innov. 2023;2:100171. 37384154 10.1016/j.pecinn.2023.100171 PMC10294099

[30] Farnan JM, Snyder Sulmasy L, Worster BK, Chaudhry HJ, Rhyne JA, Arora VM, et al. Online medical professionalism: patient and public relationships: policy statement from the American College of Physicians and the Federation of State Medical boards. Ann Intern Med. 2013;158:620–7. 23579867 10.7326/0003-4819-158-8-201304160-00100

[31] Ramasamy A, Dugyala P, Mohan C. Mobile health apps for systemic lupus erythematosus and lupus nephritis: a critical appraisal. Arthritis Res Ther. 2022;24:110. 35568874 10.1186/s13075-022-02791-0 PMC9107137

[32] El Miedany Y. e-Rheumatology: are we ready? Clin Rheumatol. 2015;34:831–7. 25708153 10.1007/s10067-015-2897-y

[33] Haque A, Cox M, Sandler RD, Hughes M. A systematic review of internet-based information on dermatomyositis and polymyositis. Int J Rheum Dis. 2020;23:1613–8. 32812386 10.1111/1756-185X.13929

[34] Househ M. The use of social media in healthcare: organizational, clinical, and patient perspectives. Stud Health Technol Inf. 2013;183:244–8. 23388291

[35] Bhatia A, Gaur PS, Zimba O, Chatterjee T, Nikiphorou E, Gupta L. The untapped potential of Instagram to facilitate rheumatology academia. Clin Rheumatol. 2022;41:861–7. 34601652 10.1007/s10067-021-05947-6 PMC8487452

[36] Naveen R, Thakare DR, Agarwal V, Aggarwal R, Gupta L. Validation of two simple patient-centered outcome measures for virtual monitoring of patients with idiopathic inflammatory myositis. Clin Rheumatol. 2022;41:765–72. 34791543 10.1007/s10067-021-05990-3 PMC8598218

[37] Afsar AP, Ghosh S, Titus RS, Cheng K, Kanawala AA, Kerkhof P, et al. Content analysis of patient support groups related to myositis on Facebook. Clin Rheumatol. 2024;43:725–32. 38212556 10.1007/s10067-023-06854-8 PMC10834555

[38] Adebesin F, Smuts H, Mawela T, Maramba G, Hattingh M. The role of Social Media in Health Misinformation and Disinformation during the COVID-19 pandemic: bibliometric analysis. JMIR Infodemiology. 2023;3:e48620. 37728981 10.2196/48620 PMC10551800

[39] Gaur PS, Gupta L. Social Media for Scholarly Communication in Central Asia and its neighbouring countries. J Korean Med Sci. 2021;36:e36. 33496088 10.3346/jkms.2021.36.e36 PMC7834904

[40] Ganatra K, Gasparyan AY, Gupta L. Modern Health Journalism and the Impact of Social Media. J Korean Med Sci. 2021;36:e162. 34100565 10.3346/jkms.2021.36.e162 PMC8185127

[41] Joshi M, Gupta L. Preparing infographics for Post-publication Promotion of Research on Social Media. J Korean Med Sci. 2021;36:e41. 33527783 10.3346/jkms.2021.36.e41 PMC7850859

[42] Traboco L, Pandian H, Nikiphorou E, Gupta L. Designing infographics: Visual Representations for Enhancing Education, Communication, and Scientific Research. J Korean Med Sci. 2022;37:e214. 35818705 10.3346/jkms.2022.37.e214 PMC9274103

[43] Gupta L, Gasparyan AY, Misra DP, Agarwal V, Zimba O, Yessirkepov M. Information and misinformation on COVID-19: a cross-sectional survey study. J Korean Med Sci. 2020;35:e256. 32657090 10.3346/jkms.2020.35.e256 PMC7358067

[44] Fazal ZZ, Sen P, Joshi M, Ravichandran N, Lilleker JB, Agarwal V, et al. COVAD survey 2 long-term outcomes: unmet need and protocol. Rheumatol Int. 2022;42:2151–8. 35964271 10.1007/s00296-022-05157-6 PMC9376047

[45] Hoff LS, Ravichandran N, Shinjo SK, Day J, Sen P, Junior JG, et al. COVID-19 severity and vaccine breakthrough infections in idiopathic inflammatory myopathies, other systemic autoimmune and inflammatory diseases, and healthy controls: a multicenter cross-sectional study from the COVID-19 vaccination in Autoimmune diseases (COVAD) survey. Rheumatol Int. 2023;43:47–58. 36271958 10.1007/s00296-022-05229-7 PMC9589602

[46] Naveen R, Parodis I, Joshi M, Sen P, Lindblom J, Agarwal V, et al. COVID-19 vaccination in autoimmune diseases (COVAD) study: vaccine safety and tolerance in rheumatoid arthritis. Rheumatology (Oxford). 2023;62:2366–76. 36315075 10.1093/rheumatology/keac624

[47] Sen RN, Griger P, Day Z, Joshi J, Nune M. Flares in IIMs and the timeline following COVID-19 vaccination: a combined analysis of the COVAD-1 and – 2 surveys. Rheumatology (Oxford). 2024;63:127–39. 37084267 10.1093/rheumatology/kead180

[48] Yoshida A, Kim M, Kuwana M, Ravichandran N, Makol A, Sen P, et al. Impaired physical function in patients with idiopathic inflammatory myopathies: results from the multicentre COVAD patient-reported e-survey. Rheumatology (Oxford). 2023;62:1204–15. 35920795 10.1093/rheumatology/keac441 PMC9384667

[49] Grignaschi S, Kim M, Zanframundo G, Ravichandran N, Lilleker JB, Sen P, et al. High fatigue scores in patients with idiopathic inflammatory myopathies: a multigroup comparative study from the COVAD e-survey. Rheumatol Int. 2023;43:1637–49. 37314497 10.1007/s00296-023-05344-z PMC10265550

[50] Nikiphorou E, Santos EJF, Marques A, Böhm P, Bijlsma JW, Daien CI, et al. 2021 EULAR recommendations for the implementation of self-management strategies in patients with inflammatory arthritis. Ann Rheum Dis. 2021;80:1278–85. 33962964 10.1136/annrheumdis-2021-220249 PMC8458093

[51] Gasparyan AY, Ayvazyan L, Blackmore H, Kitas GD. Writing a narrative biomedical review: considerations for authors, peer reviewers, and editors. Rheumatol Int. 2011;31:1409–17. 21800117 10.1007/s00296-011-1999-3

[52] Mago A, Aggarwal V, Gupta L. Telerheumatology and its interplay with patient-initiated care. Rheumatol Int. 2021;41:1883–4. 34165605 10.1007/s00296-021-04930-3 PMC8223232

[53] Naveen R, Sundaram TG, Agarwal V, Gupta L. Teleconsultation experience with the idiopathic inflammatory myopathies: a prospective observational cohort study during the COVID-19 pandemic. Rheumatol Int. 2021;41:67–76. 33150493 10.1007/s00296-020-04737-8 PMC7640991

[54] Ventola CL. Social media and health care professionals: benefits, risks, and best practices. P T. 2014;39:491–520. 25083128 PMC4103576

[55] Madanian S, Nakarada-Kordic I, Reay S, Chetty T. Patients’ perspectives on digital health tools. PEC Innov. 2023;2. 10.1016/j.pecinn.2023.100171 PMC10294099 37384154

[56] Solomon DH, Rudin RS. Digital health technologies: opportunities and challenges in rheumatology. Nat Reviews Rheumatol 2020. 2020;16:9. 10.1038/s41584-020-0461-x 32709998

[57] Nikiphorou E, Santos EJF, Marques A, Böhm P, Bijlsma JW, Daien CI et al. 2021 EULAR recommendations for the implementation of self-management strategies in patients with inflammatory arthritis. Ann Rheum Dis. 2021;0. 10.1136/annrheumdis-2021-220249 PMC8458093 33962964

[58] De Thurah A, Bosch P, Marques A, Meissner Y, Mukhtyar CB, Knitza J, et al. EULAR points to consider for remote care in rheumatic and musculoskeletal diseases. Ann Rheum Dis. 2022;81:1065–71. 35470160 10.1136/annrheumdis-2022-222341

[59] Hider S, Muller S, Gray L, Manning F, Brooks M, Heining D, et al. Digital exclusion as a potential cause of inequalities in access to care: a survey in people with inflammatory rheumatic diseases. Rheumatol Adv Pract. 2022;7:109. 10.1093/rap/rkac109 PMC9831060 36632437

[60] Al Shamsi H, Almutairi AG, Al Mashrafi S, Al Kalbani T. Implications of Language Barriers for Healthcare: a systematic review. Oman Med J. 2020;35:e122. 32411417 10.5001/omj.2020.40 PMC7201401

[61] Ajayi Sotubo O. A perspective on health inequalities in BAME communities and how to improve access to primary care. Future Healthc J. 2021;8:36–9. 33791458 10.7861/fhj.2020-0217 PMC8004339

[62] Safari R, Jackson J, Sheffield D. Digital Self-Management interventions for people with osteoarthritis: systematic review with Meta-analysis. J Med Internet Res. 2020;22:e15365. 32706657 10.2196/15365 PMC7428148

[63] Saketkoo LA, Valenzuela A, Kim S, McCann LJ, Lood C, Wahezi DM, et al. Moving forward together: collaborative landscapes of research in idiopathic inflammatory myopathies and calcinosis. Rheumatology (Oxford). 2024;63:1189–91. 37449887 10.1093/rheumatology/kead331

