# Supplementary material for: Digital Health and Self-Management in Idiopathic Inflammatory Myopathies: A Missed Opportunity?
Source: Curr Rheumatol Rep. 2024 Aug 8;26(11):383–91. doi: 10.1007/s11926-024-01157-6 (PMC11469974; doi:10.1007/s11926-024-01157-6)
Supplement: Supplementary file 1 — Supplementary Material 1 [file 11926_2024_1157_MOESM1_ESM.docx]

**Supplementary Material 2 Full texts excluded with reason**

| **Authors** | **Paper** | **Reasons for Exclusion** |
| --- | --- | --- |
| Brisca G et al. (2021) | Management and outcome of benign acute childhood myositis in pediatric emergency department | Wrong Intervention |
| Sharma et al. (2017) | Study of long-term outcome of children with juvenile dermatomyositis from a single-centre in north India | Wrong Study Design |
| Garand et al. (2023) | Current status of clinical outcome measures in inclusion body myositis: a systematised review | Wrong Study Design |
| Nickles et al. (2021) | Readability of online patient education materials for juvenile dermatomyositis | Wrong Study Design |
| Naveen et al. (2021) | Teleconsultation experience with the idiopathic inflammatory myopathies: a prospective observational cohort study during the COVID-19 pandemic | Wrong Intervention |
| Alexanderson et al. (2018) | Muscle Strength and Muscle Endurance During the First Year of Treatment of Polymyositis and Dermatomyositis: A Prospective Study | Wrong Intervention |
| Joshi et al. (2023) | Assessment of quality and reliability of YouTube videos for patient and physician education on inflammatory myositis | Wrong Intervention |
| Ramdharry et al. (2022) | Exercise in myositis: What is important, the prescription or the person? | Wrong Study Design |
| Alexanderson et al. (2014) | Resistive home exercise in patients with recent-onset polymyositis and dermatomyositis -- a randomized controlled single-blinded study with a 2-year followup | Wrong Intervention |
| Harris-Love et al. (2015) | Reliability of the adult myopathy assessment tool in individuals with myositis | Wrong Intervention |
| Gupta et al. (2023) | Addressing the unmet need for self-management strategies in idiopathic inflammatory myositis | Wrong study design |
| Sontheimer RD (2004) | The management of dermatomyositis: current treatment options | Wrong study design |
| Kobayashi et al (2020) | Clinical practice guidance for juvenile dermatomyositis (JDM) 2018-Update | Wrong study design |
| Boros et al. (2022) | Juvenile Dermatomyositis: what comes next? Long-term outcomes in childhood myositis from a patient perspective. | Wrong study design |
| Zhou et al. (2021) | A survey of treatment satisfaction with intravenous immunoglobulin among patients with inflammatory myositis | Wrong intervention |
| Kernich et al. (2008) | Patient education pages. Polymyositis | Wrong Study Design |
