# Supplementary material for: Digital Health and Self-Management in Idiopathic Inflammatory Myopathies: A Missed Opportunity?
Source: Curr Rheumatol Rep. 2024 Aug 8;26(11):383–91. doi: 10.1007/s11926-024-01157-6 (PMC11469974; doi:10.1007/s11926-024-01157-6)
Supplement: Supplementary file 2 — Supplementary Material 2 [file 11926_2024_1157_MOESM2_ESM.docx]

**Research string – Self Management in Myositis**

**Pubmed**

| Myositis (#1) | Self-Management (#2) |
| --- | --- |
| myositis OR dermatomyositis OR polymyositis OR inclusion body myositis OR idiopathic inflammatory myopathy OR autoimmune myopathy OR idiopathic inflammatory myopathies OR autoimmune myopathies OR Juvenile dermatomyositis OR myopathy OR “Muscle dystrophy” OR "Myositis"[Mesh] | Patient Education OR Self Management OR self-management OR Empowerment OR self-care OR self care OR self-regulation OR self regulation OR counselling OR counseling OR “motivational interview” OR “behavioural change technique*” OR “behaviour change” OR “behavioral change technique*” OR “behavior change” OR "Patient Education as Topic"[Mesh] OR "Patient Education Handout" [Publication Type] OR "Models, Educational"[Mesh] OR "Self Care"[Mesh] OR "Shared Medical Appointments"[Mesh] OR "Self-Management"[Mesh] OR "Motivational Interviewing"[Mesh] OR "Counseling"[Mesh] |

Hits from January 2000 to June 2023 (#1 AND #2) 🡪 3,974

**Embase**

| Myositis (#1) | Self-Management (#2) |
| --- | --- |
| ‘myositis’/exp OR ‘myositis’ OR ‘dermatomyositis’ OR ‘polymyositis’ OR ‘inclusion body myositis’ OR ‘idiopathic inflammatory myopathy’ OR ‘idiopathic inflammatory myopathies’ OR ‘autoimmune myopathies’ OR ‘juvenile dermatomyositis’ OR ‘myopathy’ OR ‘muscle dystrophy’ | ‘patient education’ OR ‘self management’ OR ‘self-management’ OR ‘empowerment’ OR ‘self-care’ OR ‘self-care’ OR ‘self regulation’ OR ‘self-regulation’ OR ‘counselling’ OR ‘counseling’ OR ‘motivational interview’ OR ‘behavioural change technique’ OR ‘behavioural change techniques’ OR ‘behaviour change’ OR ‘patient education’/exp OR ‘models, education’ OR ‘models, education’/exp OR ‘self care’/exp OR ‘shared medical appointments’/exp OR ‘self-management’/exp OR ‘motivational interviewing’/exp OR ‘counseling’/exp |

Hits from January 2000 to July 2023 (#1) and (#2) 🡪 1,179
